# A potential tumor suppressor role of PLK2 in glioblastoma

**DOI:** 10.1002/2211-5463.70000

**Published:** 2025-02-10

**Authors:** Xiangping Xia, Peirui Wang, Hua Xiao, Qishan Ran, Yan Li, Shengtao Yao

**Affiliations:** ^1^ Soochow University Medical College Suzhou China; ^2^ Department of Neurosurgery Affiliated Hospital of Zunyi Medical University China; ^3^ School of Public Health, Zunyi Medical University China

**Keywords:** CGGA, glioblastoma, polo‐like kinase, TCGA, tumor suppressor

## Abstract

Glioblastoma (GBM) is a highly malignant brain tumor with limited treatment options. Polo‐like kinase 2 (*PLK2*), a member of the polo‐like kinase family, has been variably implicated in cancer, but its role in GBM has not been fully elucidated. We utilized RNA‐seq data from multiple databases, including Gene Expression Omnibus (GEO), the Cancer Genome Atlas (TCGA), and the Chinese Glioma Genome Atlas (CGGA), and conducted experiments on human glioma cell lines to explore *PLK2*'s expression and function. The effects of PLK2 overexpression on GBM cell viability, proliferation, migration, cell cycle, and apoptosis were assessed, and the tumorigenic potential of *PLK2* was evaluated in a mouse model. *PLK2* was consistently downregulated in GBM tissues compared to normal brain tissues across several datasets. Overexpression of *PLK2* in GBM cell lines U87MG and U251 reduced their tumorigenic potential and enhanced cell cycle arrest and apoptosis, with significant reductions observed in apoptosis markers. Our findings suggest that *PLK2* may potentially function as a tumor suppressor in GBM. Hence, *PLK2* overexpression could potentially be leveraged as a therapeutic strategy to inhibit tumor progression and enhance apoptosis, providing new avenues for GBM treatment.

## Graphical abstract

*PLK2* was consistently downregulated in GBM tissues. Overexpression of *PLK2* in GBM cell lines U87MG and U251 reduced their tumorigenic potential and enhanced cell cycle arrest and apoptosis. Suggesting that *PLK2* overexpression could potentially be leveraged as a therapeutic strategy to inhibit tumor progression and enhance apoptosis, providing new avenues for GBM treatment.

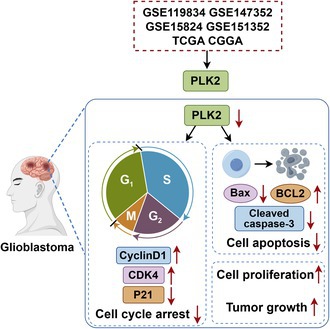

**Abbreviations**
ATCC: American Type Culture Collection
BAX: BCL2 associated X, apoptosis regulator
BCL‐2: B‐cell lymphoma 2
CCK‐8: cell counting kit‐8
CGGA: Chinese Glioma Genome Atlas
DMEM: Dulbecco's modified Eagle medium
*GAPDH*
: glyceraldehyde 3‐phosphate dehydrogenase
GBM: glioblastoma
GEO: Gene Expression Omnibus
HRP: horseradish peroxidase
NC: normal control
OE: overexpression
PI: propidium iodide
*PLK2*
: polo‐like kinase 2
PVDF: polyvinylidene fluoride
qRT‐PCR: quantitative real‐time PCR
RNA‐seq: RNA sequencing
SDS/PAGE: sodium dodecyl sulfate‐polyacrylamide gel electrophoresis
TCGA: The Cancer Genome Atlas

Glioblastoma (GBM), the most aggressive primary brain tumor, remains a significant health challenge worldwide due to its high morbidity and mortality rates [1]. According to the Central Brain Tumor Registry of the United States, glioblastomas represent about 14.2% of all primary brain tumors and 50.9% of all malignant tumors, with an incidence rate significantly higher in males than females [2]. These tumors disproportionately affect the elderly, typically diagnosed in those over 60 years old, with a median survival time of only 12–18 months following standard treatments including surgical resection, radiotherapy, and chemotherapy [3]. These ongoing developments highlight the critical need for continued research into both novel diagnostic markers and therapeutic strategies to better manage and eventually cure GBM.

Polo‐like kinase 2 (*PLK2*) is a member of the polo‐like kinase family, which plays crucial roles in cell cycle regulation and has been implicated in various cancers [4]. In colorectal cancer, high expression of *PLK2* is associated with chemoresistance, suggesting a pro‐tumorigenic role [5]. However, in breast cancer, a study suggests that PLK2 acts as a tumor suppressor in breast cancer and that PLK2 deficiency may serve as a potential biomarker [6]. In GBM, low expression or hypermethylation of PLK2 is associated with better prognosis, emphasizing that *PLK2* can be considered an independent biomarker of good prognosis in GBM [7]. Whereas another study on GBM showed that *PLK2* was downregulated in GBM, elevated *PLK2* was positively correlated with chemosensitivity and favorable prognosis in GBM patients, emphasizing that PLK2 exerts a tumor suppressor function in GBM [8]. In addition, in acute leukemia and lung cancer, *PLK2*'s function can either support cancer progression or act as a tumor suppressor depending on its expression levels and the specific cancer context [9, 10]. Thus, the role of *PLK2* in cancer can vary significantly, functioning as either an oncogene or a tumor suppressor depending on the cellular and genetic context within different tumors.

This study analyzed the expression and function of PLK2 in GBM using data from the Chinese Glioma Genome Atlas (CGGA), the Cancer Genome Atlas (TCGA), and Gene Expression Omnibus (GEO) databases. Additionally, *in vitro* functional experiments and *in vivo* studies were conducted to explore the negative regulatory role of PLK2 in GBM.

## Methods

### Data source

We selected and downloaded four raw RNA‐seq data from the GEO (https://www.ncbi.nlm.nih.gov/geo/). GSE119834 contained 44 glioblastoma stem cell (GSCs) models, 50 primary glioblastomas, and 10 neural stem cells (NSCs); GSE147352 contained 85 adult glioblastomas, 18 lower grade gliomas, and 15 normal brain tissues; GSE15824 contained 12 primary glioblastomas; GSE151352 contained normal/tumor tissue pairs of 12 GBM patients. We also obtained RNA‐seq data from 169 GBM tissue samples and 5 normal samples from the TCGA database, as well as RNA‐seq data from 321 GBM patients from the CGGA database.

### PLK2 expression analyses

After quality control [11], RNA‐seq data were analyzed by r package edger [12] and limma package [13] was used to calculate and normalize the obtained expression data of PLK2.

### Collection of clinical samples

In this experiment, a total of 35 clinical samples were collected from GBM patients, including both tumor tissues and adjacent noncancerous tissues. Due to variations in tissue quality, only 30 samples were included in the study. After surgery, these samples were stored at −80 °C for subsequent RNA extraction and protein collection. All patients or their family members signed written informed consent forms. This study was followed the Declaration of Helsinki and approved by the ethics committee of Affiliated Hospital of Zunyi Medical University with approval number: KLL‐2023‐589.

### Cell culture

Human astrocyte cell line SVG p12 and five human glioma cell lines (U87MG, U251, LN229, T98G, and A172) were obtained from the American Type Culture Collection (ATCC, Manassas, VA, USA). Cells were cultured in Dulbecco's modified Eagle medium (DMEM) supplemented with 10% fetal bovine serum (FBS) (#10099‐141; Gibco, Carlsbad, CA, USA) and 1% penicillin–streptomycin solution. The cultures were maintained in a 37 °C incubator with 5% CO_2_ atmosphere. Passaging was performed when cell confluence reached 80–90%.

### Cell transfection

Cell transfection was conducted using Lipofectamine 3000 (#L3000015; Invitrogen, Carlsbad, CA, USA). The PLK2 overexpression vector, named pCMV‐PLK2, was used to facilitate the overexpression of the PLK2 gene. This vector and the corresponding reagents were purchased from GenScript (#OHu22350D, Piscataway, NJ, USA). U87MG and U251 human glioma cells were seeded at a density of 1 × 10^6^ cells per well in 6‐well plates. Transfection was carried out when the cell density reached 50–70%. Approximately 48 h post‐transfection, the cells were harvested for further analysis. The success of the transfection was verified using quantitative real‐time PCR (qRT‐PCR).

### qRT‐PCR

Total RNA was extracted from clinical samples and cell lines using the RNeasy Mini kit (#74104; QIAGEN, Hilden, Germany) according to the manufacturer's instructions. The quality and quantity of the isolated RNA were assessed before proceeding. Reverse transcription was performed using the High‐Capacity cDNA Reverse Transcription kit (#4368814; Thermo Fisher Scientific, Waltham, MA, USA). The obtained complementary DNA served as a template for further PCR amplification using SYBR Green intercalating dye (#163795‐75‐3; Merck KGaA, Darmstadt, Germany). $2^{-\Delta \Delta C_{t}}$ method was employed to analyze the resulting data. *GAPDH* was used as the reference gene, and expression levels of *PLK2*, *cyclin D1*, *CDK4*, and *P21* were normalized to *GAPDH*. Primer sequences were listed in Table 1.

**Table 1:** Primer sequences of the target genes.

Genes	Primer	Primer sequences
*PLK2*	Forward	5′‐CTACGCCGCAAAAATTATTCCTC‐3′
	Reverse	5’‐TCTTTGTCCTCGAAGTAGTGGT‐3’
*Cyclin D1*	Forward	5′‐CCGTCCATGCGGAAGATC‐3′
	Reverse	5′‐GTCACACTTGATCACTCTGG‐3′
*CDK4*	Forward	5′‐CGGAGCTGAAGCTCAAGG‐3′
	Reverse	5′‐GCGGCTTCTTCATCTTCA‐3′
*P21*	Forward	5′‐AGGTGGACCTGGAGACTCTCAG‐3'
	Reverse	5'‐TCCTCTTGGAGAAGATCAGCCG‐3′
*GAPDH*	Forward	5′‐AGAAGGCTGGGGCTCATTTG‐3′
	Reverse	5′‐AGGGGCCATCCACAGTCTTC‐3′

### Western blot

Total proteins from clinical samples and cell lines were extracted and quantified before SDS/PAGE separation and PVDF membrane transfer. The membrane was blocked with 10% milk in TBST and incubated overnight at 4 °C with primary antibodies: anti‐PLK2 (#ab137539, 1 : 1000; Abcam, Cambridge, UK), anti‐cleaved caspase‐3 (#ab32042, 1 : 500; Abcam), anti‐BAX (#ab182733, 1 : 2000; Abcam), anti‐BCL‐2 (#ab182858, 1 : 2000; Abcam), and anti‐β‐actin (#ab8226, 1 : 1000; Abcam). Following TBST washes, secondary antibodies Goat Anti‐Mouse IgG H&L (HRP) (#ab205719, 1 : 2000; Abcam) and Goat Anti‐Rabbit IgG H&L (HRP) (#ab205718, 1 : 2000; Abcam) were applied for 1 h at room temperature. The membrane was washed and developed using a Bio‐Rad, Hercules, CA, USA detection system.

### CCK‐8 assay

U87MG, U251, U87MG^OE‐PLK2^, and U251^OE‐PLK2^ were seeded into a 96‐well plate. After culturing for 1, 2, 3, and 4 days, CCK‐8 reagent (#CK04; Dojindo, Shanghai, China) was added to the wells. Following incubation with the CCK‐8 reagent, the absorbance of the wells was measured at 490 nm using a microplate reader (Multiskan GO, Thermo Fisher).

### Cell colony formation

After counting, U87MG, U251, U87MG^OE‐PLK2^, and U251^OE‐PLK2^ were seeded into a 6‐well plate and cultured in a 37 °C incubator with 5% CO_2_ to form colonies. The cells were then fixed and stained with crystal violet to visualize the colonies. Finally, the colonies were counted and analyzed.

### Transwell assay

After counting, U87MG, U251, U87MG^OE‐PLK2^, and U251^OE‐PLK2^ were seeded into the upper chambers (#PEZGS0416; Merck Millipore) with FBS‐free DMEM medium and cultured in a 37 °C incubator with 5% CO_2_. Complete medium was added to the lower transwell chambers. The cells in the upper chamber were wiped with a cotton swab. Cells in the lower chambers were fixed with 4% paraformaldehyde for 15 min and stained with crystal violet for 10 min. Several fields were randomly selected under the microscope (Nikon Eclipse Ti2, chiyoda‐ku, Tokyo, Japan) for imaging and counting.

### Wound‐healing assay

After counting, U87MG, U251, U87MG^OE‐PLK2^, and U251^OE‐PLK2^ were seeded into a 6‐well plate and cultured in a 37 °C incubator with 5% CO_2_ for 24 h, allowing the formation of a confluent layer. Then, a ‘wound’ was created in the cell monolayer using a pipette tip. Next, replace the culture medium and put the cells to original incubator for 48 h. Notably, images of the wound should be captured at the beginning and end of incubation for calculating the wound‐healing percentage.

### Flow cytometry for cell cycle assay

After culturing the cells to the logarithmic growth phase, they were washed with PBS and digested with 0.25% trypsin–EDTA. The cells were collected and fixed with 70% ethanol. Following fixation, 50 μg·mL^−1^ propidium iodide (PI, #P4864; Sigma, Riedstrasse, Germany) and 100 μg·mL^−1^ RNase A (#R6513; Sigma) were added and incubated at 37 °C for 30 min in the dark. The cell cycle distribution was analyzed by BD FACSCanto II Flow Cytometer (BD Biosciences, San Jose, CA, USA), and the data were processed with flowjo software (v10.6), Ashland, OR, USA.

### Cell apoptosis

Glioblastoma cells were collected and stained with Annexin V‐FITC/PI according to the instruction of Annexin V‐FITC/PI Apoptosis Detection Kit (#ab14085; Abcam). The fluorescence intensity was detected by BD FACSCanto II Flow Cytometer, and the data analysis was performed by flowjo software.

### Subcutaneous tumor mouse model

BALB/c nude mice, aged 6 weeks, were acquired from the Laboratory Animal Center of Yunnan University. The mice were kept in animal facilities of SPF‐grade, with a temperature of 24 ± 1 °C, humidity between 50% and 60%, and a light/dark cycle of 12 h. The housing and experimental procedures followed the National Institutes of Health Guidelines for the Care and Utilization of Laboratory Animals. Approval for the research was granted by Biomedical Research Ethics Committee of Affiliated Hospital of Zunyi Medical University, with the assigned approval number KLL‐2023‐589. Each group of 5 BALB/c nude mice was injected subcutaneously with around 5 × 10^6^ cells (*n* = 5). The weight of each mouse and the size of each tumor were measured using a vernier caliper every 3 days to monitor changes in tumor volume. The tumor volume was calculated by *V* = (*W* ^2^ × *L*)/2, where *V* is tumor volume, *W* is tumor width, and *L* is tumor length. The mice were euthanized after 18 days by inhaling excessive isoflurane in a quiet environment.

### Statistical analysis

graphpad prism version 9.5.1, GraphPad Software, San Diego, CA, USA was used to perform statistical analysis. All experiments were repeated at least three times, and the results were presented as mean ± SD. Student's *t* test was used to evaluate significance (*P* < 0.05) between two groups. For analyses involving two independent variables, such as treatment groups and time points, two‐way ANOVA was performed to evaluate the effects of both factors and their interaction on tumor volume or other outcomes.

## Results

### PLK2 showed lower expression in GBM

**Fig. 1:**
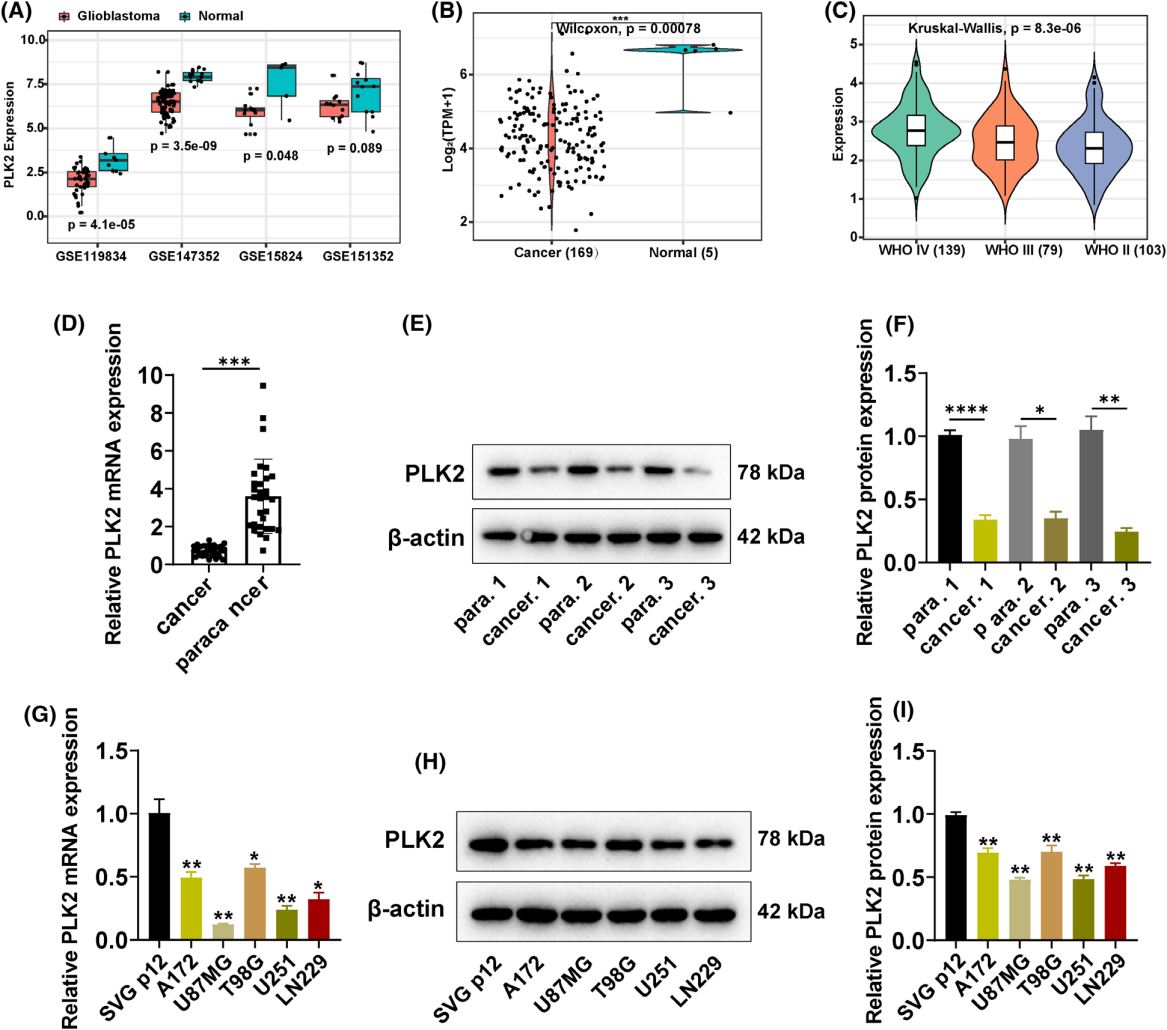
PLK2 showed lower expression in glioblastoma. (A) The expression level of *PLK2* in four GEO datasets. (B) The expression level of *PLK2* in TCGA database. Cancer (*n* = 169), Normal (*n* = 5). (C) The expression level of PLK2 in GBM patients at stages II, stage III, or stage IV. WHO IV (*n* = 139), WHO III (*n* = 79), WHO II (*n* = 103). (D) mRNA and (E) protein levels of *PLK3* in 30 glioma patients' cancer tissue samples and their adjacent normal tissue samples. (F) Statistical analysis of (E). (G) mRNA and (H) protein levels of *PLK3* in human glioma cell lines U87MG, U251, LN229, T98G, and A172. (I) Statistical analysis of (H). *n* = 3, *P ≤ 0.05; **P ≤ 0.05; ***P ≤ 0.05. Para: adjacent normal tissue sample. Error bars indicate SD.

To investigate the expression of *PLK2* in GBM, we first analyzed the levels of PLK2 in normal and tumor tissues of GBM patients from four GEO datasets. As shown in Fig. 1A, the expression of *PLK2* in tumor tissues was significantly lower than in normal tissues, with a statistically significant difference (*P* < 0.05). Furthermore, we validated these results by analyzing RNA‐seq data of 169 GBM tissue samples and 5 normal samples from the TCGA database. Here, *PLK2* exhibited the same downregulated expression trend in tumor samples (Fig. 1B). Gliomas are classified into four grades based on their malignancy: Grades I and II are low‐grade well‐differentiated gliomas with lower malignancy, Grade III represents anaplastic gliomas, and Grade IV corresponds to glioblastoma. Grades III and IV gliomas are both poorly differentiated and highly malignant. Next, we compared the expression of the PLK2 in GBM patients at different stages from the CGGA database. It can be observed that the expression level of *PLK2* upregulated as the malignancy of gliomas increased (Fig. 1C). One possible explanation is that the low expression of *PLK2* plays a more critical role in the early stages of glioma development. However, as the tumor progresses to higher grades, other potent oncogenic factors may drive malignant progression to a greater extent, thereby diminishing the impact of *PLK2*.

Thirty glioma patients' cancer tissue samples and their adjacent normal tissue samples were collected. The total RNA and protein were extracted for qRT‐PCR and western blot to validate the clinical expression levels of *PLK2*. Consistent with the results of the database analysis, the mRNA and protein levels of PLK2 in GBM tissues were significantly lower than in adjacent normal tissues (Fig. 1D–F). This phenomenon was also observed in five different common types of human glioma cell lines U87MG, U251, LN229, T98G, and A172, where SVGp12 cells, a normal human glial cell line, served as a control [14] (Fig. 1G–I). Above results together indicated the inhibition of *PLK2* expression in GBM tissues, which may link with GBM origin.

### PLK2 negatively regulated GBM cell proliferation and migration

**Fig. 2:**
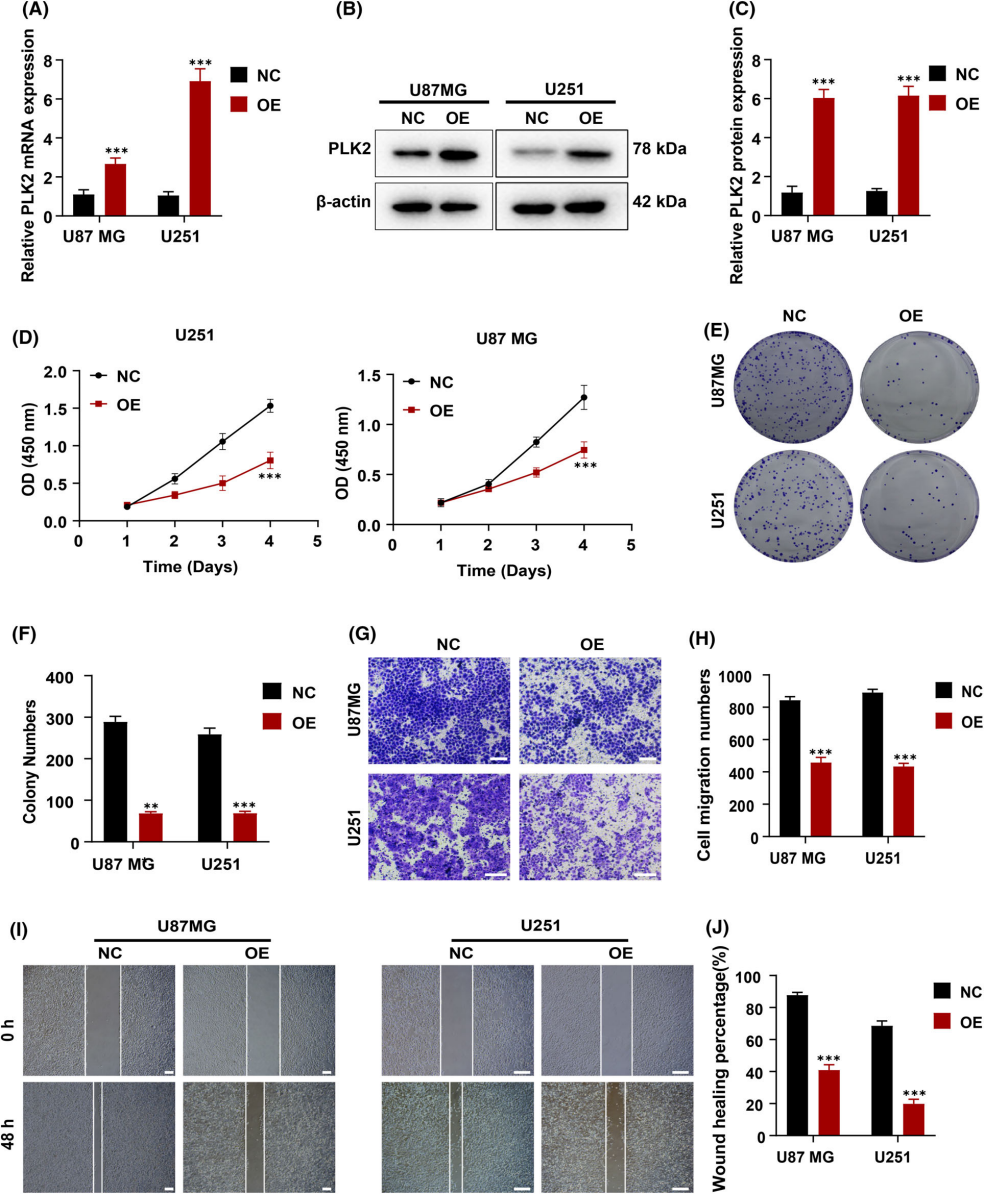
*PLK2* negatively regulated GBM cell proliferation and migration. (A) mRNA and (B) protein levels of *PLK3* in U87MG, U251, U87MG^OE‐PLK2^, and U251^OE‐PLK2^. (C) Statistical analysis of B. (D) Cell viability of U87MG, U251 U87MG^OE‐PLK2^, and U251^OE‐PLK2^. (E) Results of colony formation assay. (F) Statistical analysis of E. (G) Results of Transwell assay. Scale bar = 100 μm. (H) Statistical analysis of G. (I) Results of wound‐healing assay. Scale bar = 100 μm. (J) Statistical analysis of (I). *n* = 3 biologically independent samples. Student's *t* test or one‐way ANOVA was applied to analyze the statistical significance. **P ≤ 0.01; ***P ≤ 0.001. NC, cell expressed *PLK2* normally; OE, cell over‐expressed *PLK2*. Error bars indicate SD.

Since *PLK2* exhibited a downregulation trend in various databases, clinical samples, and human glioma cell lines, we conducted experiments by overexpressing *PLK2* (Fig. 2A–C) in glioma cell lines to investigate its biological impact. According to Fig. 1G,H, PLK2 showed the lowest expression levels in U87MG and U251. Therefore, these two cell lines were chosen for subsequent experiments. Compared with original U87MG and U251, viability of U87MG^OE‐PLK2^ and U251^OE‐PLK2^ decreased significantly (Fig. 2D). The clone formation rates of U87MG^OE‐PLK2^ and U251^OE‐PLK2^ were notably lower than those of U87MG and U251, indicating weakened proliferation after PLK2 overexpression (Fig. 2E,F). What is more, lower cell migration was expectedly observed in Transwell and wound‐healing assay results (Fig. 2G–J). Based on these experimental results, PLK2 may play a negative regulatory role in GBM cells, especially as its overexpression can reduce or inhibit the proliferation and migration ability of glioma cell lines, which holds potential significance for glioma treatment and control.

### Overexpressing of *PLK2* promoted GBM cell cycle arrest and apoptosis

**Fig. 3:**
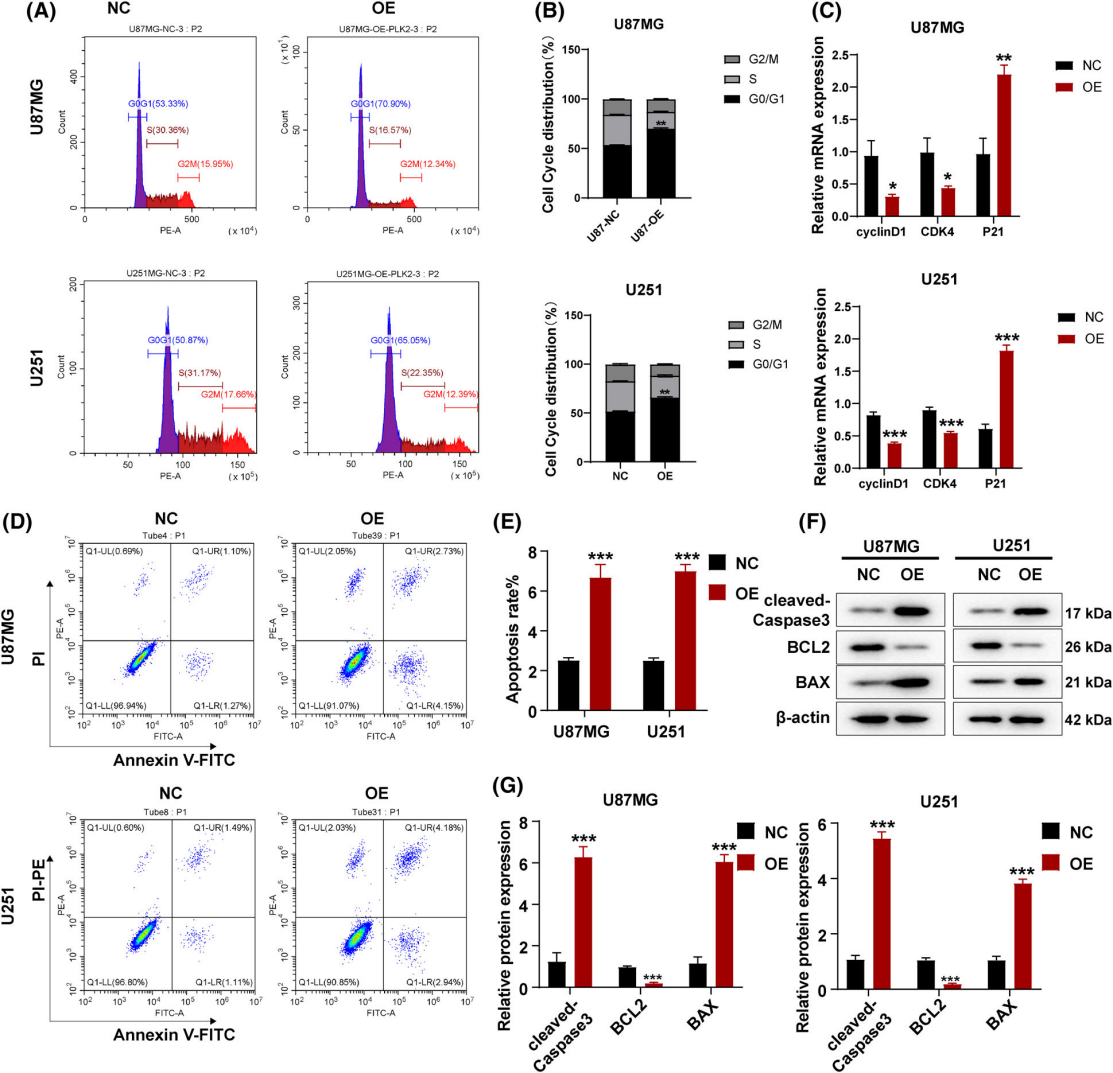
Overexpressing of *PLK2* promoted GBM cell cycle arrest and apoptosis. (A) Cell cycle analysis of U87MG and U251. (B) Quantification of the flow cytometry cell cycle assay. (C) mRNA levels of *cyclinD1, CDK4, P21* in U87MG, U251, U87MG^OE‐PLK2^, and U251^OE‐PLK2^. (D) Apoptosis detected in U87MG, U251, U87MG^OE‐PLK2^, and U251^OE‐PLK2^ using Annexin V‐FITC/PI. (E) Statistical analysis of (D). (F) Protein expression levels of cleaved caspase‐3, BAX, and BCL‐2 using western blot. (G) Statistical analysis of (F). *n* = 3 biologically independent samples. Student's *t* test or one‐way ANOVA was applied to analyze the statistical significance. *P ≤ 0.05; **P ≤ 0.01; ***P ≤ 0.001. Error bars indicate SD.

We further investigated the effect of *PLK2* on the cell cycle of GBM cells and found that U87MG^OE‐PLK2^ and U251^OE‐PLK2^ cells significantly promoted G0/G1 phase arrest in GBM cells (Fig. 3A,B). Moreover, *PLK2* overexpression significantly upregulated the mRNA expression of the cell cycle‐related gene *P21*, while inhibiting the mRNA expression of *cyclin D1* and *CDK4* (Fig. 3C). This further indicates that *PLK2* can induce cell cycle arrest in GBM cells. A previous study has reported that silencing of *PLK2* increased cell proliferation and decreased apoptosis in SGC‐7901 gastric cancer cells [15]. Here we also investigated the relationship between PLK2 overexpression and apoptosis. We found that U87MG^OE‐PLK2^ and U251^OE‐PLK2^ cells significantly increased the levels of early and late apoptosis as measured by membrane‐bound protein V and PI staining (Fig. 3D,E). Consistent with flow cytometry, the levels of apoptosis markers, cleaved caspase‐3, and pro‐apoptotic protein BAX increased, while the levels of anti‐apoptotic protein BCL‐2 decreased after *PLK2* overexpression (Fig. 3F,G), indicating the apoptosis‐promoting role of *PLK2* in GBM.

### Overexpression of *PLK2* inhibits tumor growth in mice

**Fig. 4:**
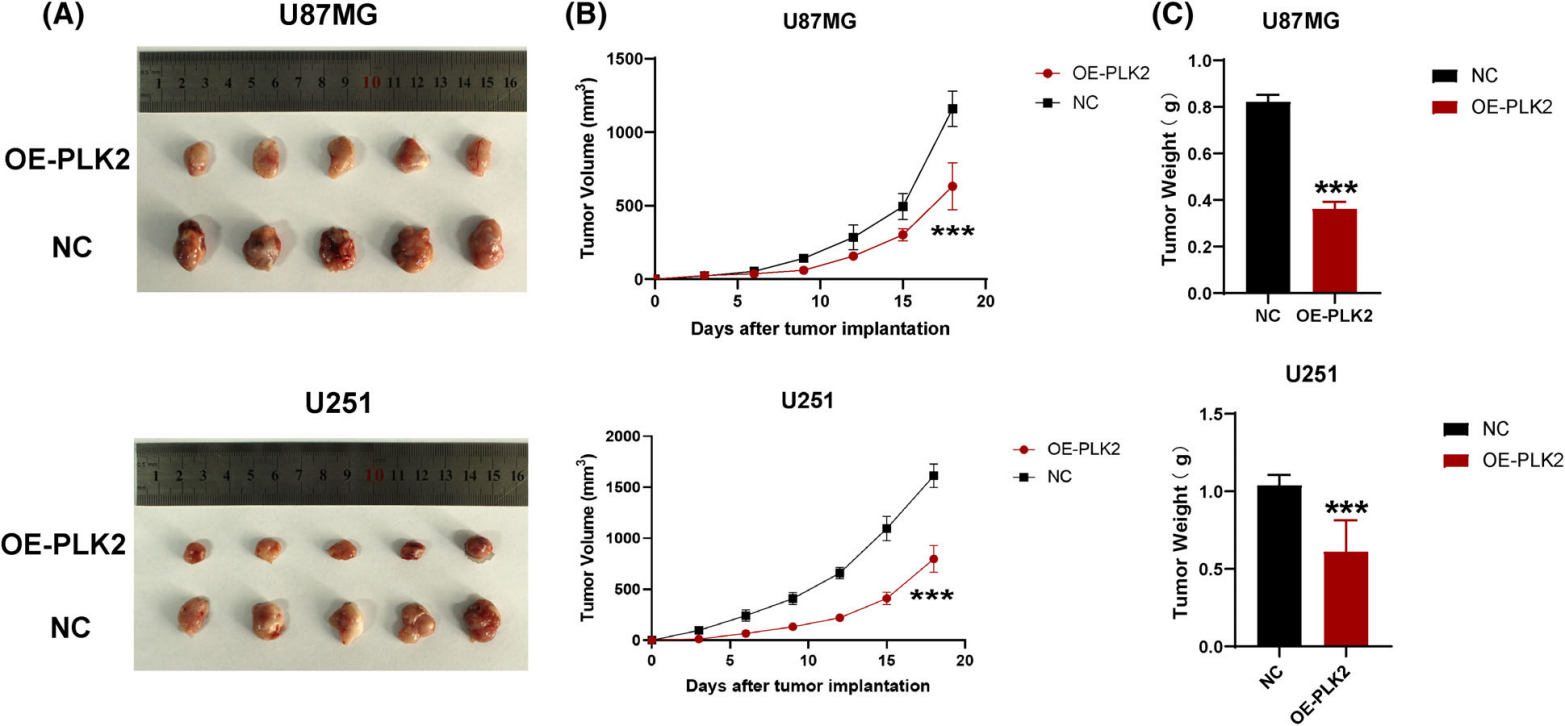
Overexpression of PLK2 inhibits tumor growth in mice. (A) Pictures of tumors in mice. (B) Tumor growth volume curves. (C) Tumor weight. *n* = 5. Two‐way ANOVA was used to evaluate tumor volume. ***P ≤ 0.001. Error bars indicate SD.

To assess the effect of *PLK2* on tumor growth *in vivo*, we established a subcutaneous GBM model in athymic nude mice using GBM cells stably transfected with OE‐PLK2. The results showed that U87MG^OE‐PLK2^ and U251^OE‐PLK2^ cells significantly inhibited tumor growth compared to controls (Fig. 4A–C).

In order to further investigate the mechanism of *PLK2* in GBM, we searched the GPSAdb database (https://www.gpsadb.com/) and found that the expression level of *PLK2* is significantly reduced when genes such as *TP53*, *PTPN11*, *JMID1C*, *CHASERR*, and *SLC1A5* are knocked out or knocked down in glioma cell lines U‐87MG and T98G. The dataset information is in Table S1. This finding suggests that *TP53*, *PTPN11*, *JMID1C*, *CHASERR*, and *SLC1A5* may serve as upstream regulatory genes of *PLK2*, which points to the direction for our next research.

## Discussion

Glioblastoma remains a formidable challenge in neuro‐oncology, characterized by a dismal prognosis and limited treatment advancements. Recent studies such as those by Rahman *et al*. [16], Obrador *et al*. [17], and Qi *et al*. [18] highlight the persistent obstacles in improving patient outcomes, including the blood–brain barrier, tumor heterogeneity, and resistance mechanisms. Innovations in therapy, such as the use of nanotherapies and oncolytic viruses as discussed by Louis *et al*. [19] and Hamad *et al*. [20], demonstrate potential in circumventing these barriers. However, the need for novel diagnostic methods and a deeper understanding of GBM's molecular pathogenesis is urgent to develop more effective treatments. The exploration of combination therapies and advancements in immunotherapy also offer hope for enhancing treatment efficacy and patient survival.

Glioblastoma studies utilizing sequencing databases like the CGGA and GEO have made significant strides in unraveling the disease's etiology. The research by Wang *et al*. on RNA editing profiles underscores emerging molecular insights, suggesting new prognostic models for lower grade gliomas [21]. Similarly, Zhao's study on endothelial cell hub genes in GBM offers a promising prognostic model integrating single‐cell and bulk RNA sequencing, highlighting diverse immunological features and prognosis [22]. Despite these advancements, many studies often lack depth in fully elucidating the underlying mechanisms, leaving potential areas for further exploration.

In recent GBM research, *PLK2* has been identified as a crucial factor with dual roles, influencing both tumor progression and therapeutic resistance. Tan *et al*. [23] reveal that DYRK1A‐mediated phosphorylation of *PLK2* promotes GBM cell proliferation, migration, and invasion, suggesting its potential as an oncogenic kinase. Similarly, Tan *et al*. [23] assess *PLK2*'s prognostic relevance, highlighting its increased expression in GBM tissues compared to normal brain tissue, which correlates with poor patient outcomes, reinforcing its role in tumor aggressiveness. Cao *et al*. [24] also demonstrated that knocking down *PLK2* reduces cell proliferation and induces apoptosis in glioma cells, suggesting that *PLK2* functions as an oncogene. Conversely, the study by Alafate *et al*. [8] presents a different perspective. Alafate *et al*. find that loss of *PLK2* induces resistance to the chemotherapeutic agent temozolomide through activation of the Notch signaling pathway, complicating its role in GBM pathogenesis and therapy resistance. The pathogenic mechanisms of *PLK2* in GBM remain not fully understood, necessitating further exploration to elucidate its intricate roles in tumor biology and response to therapy.

In this study, we systematically analyzed *PLK2* expression across multiple datasets and clinical samples, uncovering a significant reduction of *PLK2* in GBM tissues compared to normal tissues. This trend persisted across the GEO and TCGA databases as well as in clinical validations using qRT‐PCR and western blot analyses. Functional experiments revealed that overexpression of *PLK2* in glioma cell lines U87MG and U251 resulted in decreased cell proliferation, colony formation, and migration, suggesting that *PLK2* acts as a tumor suppressor in GBM. Moreover, *PLK2* overexpression significantly increased cell cycle arrest and apoptosis in GBM cells and inhibited subcutaneous tumor growth. These findings suggest that enhancing *PLK2* expression could be a potential therapeutic strategy for inhibiting GBM progression and improving patient outcomes.

This study delineates a critical role of *PLK2* in GBM, distinguishing it as a potential tumor suppressor, contrasting with prior studies which have typically focused on the oncogenic roles of other *PLK* family members in various cancers. Previous research has often highlighted the proliferative and tumorigenic effects of PLKs in malignancies like colorectal cancer and medulloblastoma, underscoring their potential as therapeutic targets. However, our findings specifically underscore *PLK2*'s suppression of GBM cell proliferation and its promotion of apoptosis, suggesting a divergent role within the PLK family that could redefine therapeutic approaches to GBM. Additionally, the use of comprehensive datasets (GEO, TCGA, CGGA) and a variety of experimental approaches enhances the reliability of these observations compared to earlier studies that may have had more limited scope or smaller sample sizes.

*PLK2* is emerging as a critical player in the molecular interactions and epigenetic mechanisms within various cancers, including GBM. Studies such as those by Cao *et al*. [24] and Alverez *et al*. [25] demonstrate *PLK2*'s role in cellular proliferation and apoptosis, highlighting its potential as a therapeutic target. Similarly, Kim *et al*. [26] provide structural insights into *PLK2*'s polo‐box domain, which is pivotal for its regulatory functions in the cell cycle. Furthermore, Ling *et al*. [27] show that the histone deacetylase *SIRT1* targets *PLK2* for regulation of centriole duplication, underscoring the enzyme's involvement in chromosomal stability. This interaction suggests a broader regulatory role that spans beyond tumor suppression, linking to the core processes of cellular division and genetic stability. Additionally, research by Xu *et al*. [28] and Zurnic *et al*. [29] illustrate *PLK2*'s involvement in intricate molecular pathways that impact drug resistance and viral interactions in cancers, emphasizing its multifaceted role in disease pathogenesis.

As we move forward, our research will continue to explore the function of *PLK2* in GBM using *in vivo* animal tumor models. Building on the GPSAdb database findings, which suggest that *TP53*, *PTPN11*, *JMID1C*, *CHERRR*, and *SLC1A5* may be involved in the downregulation of *PLK2*, we aim to delve deeper into its molecular mechanisms. This could potentially unlock new therapeutic strategies. Looking ahead, expanding this research under more diverse experimental conditions will be insightful. These efforts not only promise to enhance our understanding of *PLK2*'s role in GBM but also challenge us to reconsider treatment approaches for this complex and deadly disease.

## Conclusions

This study reveals that *PLK2* acts as a potential tumor suppressor in GBM, demonstrating a consistent pattern of reduced expression in GBM tissues compared to normal tissues. Overexpression of *PLK2* significantly inhibits GBM cell proliferation and migration, while promoting cell cycle arrest, apoptosis, and suppression of tumor growth. These findings highlight the therapeutic potential of *PLK2* in counteracting GBM progression.

## Conflict of interest

The authors declare no conflict of interest.

## Author contributions

SY and XX conceived and designed the project, XX, PW, and HX acquired the data, QR and YL analyzed and interpreted the data, XX and SY wrote the paper.

## Supporting information


**Table S1.** Information on datasets.

## Data Availability

The data utilized in this study are available in the GEO database under accession numbers GSE119834, GSE147352, GSE15824, and GSE151352. The datasets analyzed during the current study are available from the corresponding author on reasonable request.
