# Supplementary material for: A potential tumor suppressor role of PLK2 in glioblastoma
Source: FEBS Open Bio. 2025 Feb 10;15(5):856–66. doi: 10.1002/2211-5463.70000 (PMC12051019; doi:10.1002/2211-5463.70000)
Supplement: Supplementary file 1 — Table S1. Information on datasets. [file FEB4-15-856-s001.docx]

**Table S1. Information on datasets**

| **Dataset** | **pbgene** | **Cell Line** | **method** | **Gene** |
| --- | --- | --- | --- | --- |
| D23431 | PTPN11 | U87MG | shRNA | PLK2 |
| D21709 | JMJD1C | U87MG | shRNA | PLK2 |
| D21561 | TP53 | U87MG | KO | PLK2 |
| D21565 | TP53 | U87MG | KO | PLK2 |
| D25988 | CHASERR | T98G | shRNA | PLK2 |
| D25344 | SLC1A5 | T98G | shRNA | PLK2 |
